# Recommendations for the treatment of epilepsy in adult and pediatric patients in Belgium: 2020 update

**DOI:** 10.1007/s13760-020-01488-y

**Published:** 2020-10-13

**Authors:** Paul Boon, Susana Ferrao Santos, Anna C. Jansen, Lieven Lagae, Benjamin Legros, Sarah Weckhuysen

**Affiliations:** 1 Reference Center for Refractory Epilepsy, Department of Neurology, Ghent University Hospital, Ghent, Belgium; 2 Refractory Epilepsy Center, Saint-Luc University Hospital, Brussels, Belgium; 3 Pediatric Neurology Unit, Department of Pediatrics, UZ Brussel, Vrije Universiteit Brussel, Brussels, Belgium; 4 Reference Center for Refractory Epilepsy, Pediatric Neurology, Department of Development and Regeneration, University Hospitals Leuven, Leuven, Belgium; 5 Department of Neurology, Reference Center for the Treatment of Refractory Epilepsy, Hôpital Erasme, Université Libre de Bruxelles (ULB), Brussels, Belgium; 6 Department of Neurology, Antwerp University Hospital, Antwerp, Belgium; 7 VIB-Center for Molecular Neurology, University of Antwerp, Antwerp, Belgium

**Keywords:** Epilepsy, Seizures, Antiepileptic drugs, Recommendations, Monotherapy, Add-on therapy

## Abstract

To guide health care professionals in Belgium in selecting the appropriate antiepileptic drugs (AEDs) for their epilepsy patients, a group of Belgian epilepsy experts developed recommendations for AED treatment in adults and children (initial recommendations in 2008, updated in 2012). As new drugs have become available, others have been withdrawn, new indications have been approved and recommendations for pregnant women have changed, a new update was pertinent. A group of Belgian epilepsy experts (partly overlapping with the group in charge of the 2008/2012 recommendations) evaluated the most recent international guidelines and relevant literature for their applicability to the Belgian situation (registration status, reimbursement, clinical practice) and updated the recommendations for initial monotherapy in adults and children and add-on treatment in adults. Recommendations for add-on treatment in children were also included (not covered in the 2008/2012 publications). Like the 2008/2012 publications, the current update also covers other important aspects related to the management of epilepsy, including the importance of early referral in drug-resistant epilepsy, pharmacokinetic properties and tolerability of AEDs, comorbidities, specific considerations in elderly and pregnant patients, generic substitution and the rapidly evolving field of precision medicine.

## Introduction

Epilepsy is one of the most common neurological diseases, affecting approximately 50,000,000 people worldwide [1]. In Belgium, an estimated 100,000 people are living with epilepsy [2]. The disease is defined by the occurrence of at least two unprovoked seizures more than 24 h apart or one unprovoked seizure and a high risk of recurrence (at least 60%), or by the diagnosis of an epilepsy syndrome [3]. In 2017, the International League Against Epilepsy (ILAE) updated their classification of seizure types, using seizure onset as their basis. They distinguish focal-onset seizures (originating in one hemisphere, formerly called partial seizures), generalized-onset seizures (originating in both hemispheres, e.g., absences, generalized tonic–clonic, atonic and myoclonic seizures) and seizures of unknown onset [4]. Seizures that start focally and spread to bilateral tonic–clonic movements (previously called secondarily generalized tonic–clonic seizures) are referred to as focal-to-bilateral tonic–clonic seizures [4].

**Fig. 1:**
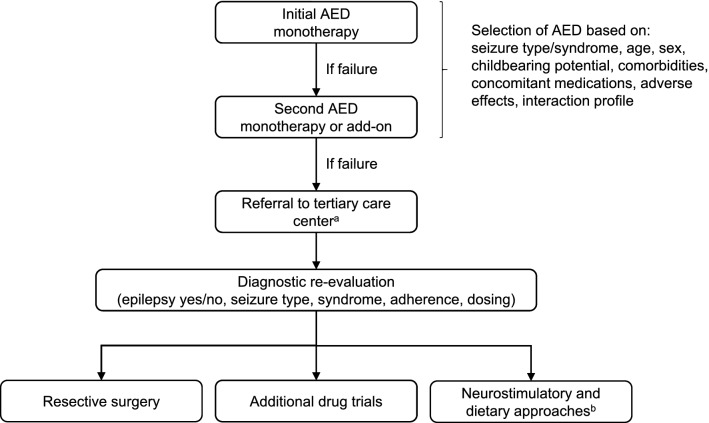
Epilepsy treatment pathway. AED, antiepileptic drug. ^a^Prompt referral of patients with drug-resistant epilepsy or certain complex epilepsy syndromes is crucial to improve a patient’s chances to achieve seizure control and avoid life-long sequelae. ^b^Neurostimulatory approaches include vagus nerve and deep brain stimulation; dietary approaches include ketogenic and modified Atkins diet

Once a diagnosis of epilepsy is confirmed, most patients are treated with antiepileptic drugs (AEDs). Currently, more than 20 different AEDs are registered in Belgium [5]. While this large number allows tailoring treatment to individual patients’ needs, it makes selection of the most suitable compound complex. The choice of an AED depends on the patient’s seizure type, age, sex, childbearing potential, comorbidities and concomitant medications, and the drug’s adverse effect and interaction profiles (Fig. 1). To guide health care professionals in Belgium in making this choice, a group of experts developed recommendations for the management of epilepsy in adults and children in general neurological practice in Belgium in 2008 [6] and updated these in 2012 [7]. Since then, new indications of previously available AEDs have been approved (e.g., monotherapy and changes in the lower age limit for lacosamide [8, 9]), new AEDs have become available (e.g., perampanel and brivaracetam [10, 11]) and others have disappeared from the market (e.g., pheneturide and retigabine [12, 13]). Changes in clinical practice are also warranted for certain subpopulations, especially pregnant women. A new update of the recommendations therefore seemed pertinent.

## Methodology

The initial recommendations for the management of epilepsy in general neurological practice in Belgium [6] and the updated recommendations [7] were based on guidelines by the ILAE [14], the American Academy of Neurology (AAN [15, 16]), the Scottish Intercollegiate Guidelines Network (SIGN [17]) and the United Kingdom National Institute for Health and Care Excellence (NICE [18]), and relevant articles on controlled clinical trials published after the cut-off dates used in these guidelines. This resulted in recommendations for initial monotherapy in adults and children and for add-on treatment in adults [6, 7].

In October 2019, a group of Belgian epilepsy experts (partly overlapping with the group in charge of the 2008/2012 recommendations) convened to discuss the strategy to update the current recommendations. Following this first meeting, the ILAE, AAN, SIGN and NICE websites were searched for updates to their guidelines. The AAN published updated guidelines for the treatment of new-onset and drug-resistant epilepsy in 2018 [19, 20], SIGN published updated guidelines for the management of epilepsy in adults in 2015 with revisions in 2018 [21] and NICE published updated guidelines for the management of epilepsy in children and adults in 2012 with a last revision in 2020 [22]. No updates to the ILAE guidelines (encompassing the general adult or pediatric patient population) were found but an updated evidence review of AED efficacy and effectiveness as initial monotherapy was published in 2013 [23], and a report on the management of epilepsy during pregnancy in 2019 [24]. As the last fully updated guidelines (AAN) were based on a systematic literature search up to November 2015, the information extracted from the different guidelines was supplemented with information from (systematic) reviews and/or meta-analyses on AED treatment (comparing multiple AEDs) published between January 2016 and February 2020, retrieved by searching PubMed and the Cochrane Library.

The experts evaluated the updated AAN, SIGN and NICE guidelines, the ILAE evidence review and the relevant published articles for their applicability to the Belgian situation (registration status, reimbursement, clinical practice) and prepared an update of the recommendations for initial monotherapy in adults and children and add-on treatment in adults. In addition, recommendations for add-on treatment in children were included, which were not covered in the 2008/2012 publications.

The following criteria were used to prepare the treatment recommendations in this update:

1. The AED is registered and reimbursed in Belgium [5, 25]. Remarks are included for drugs that were proven to be effective for a certain indication but are currently not registered or reimbursed for that indication in Belgium.
2. The AEDs with the highest level of evidence for efficacy across the different guidelines or evidence reviews are recommended as first choice. As the different guidelines do not all use the same method to rate the evidence in the studies they reviewed, no levels of evidence for efficacy are stated in the current recommendations.
3. In the rare cases where the level of evidence for different AEDs was the same or where evidence was limited, recommendations were based on a consensus among the authors.

The same definitions as in the 2008/2012 recommendations were used [6, 7]:

- *First choice*: First treatment choice in a patient without any specific factors precluding the use of this AED (e.g., comorbidity, concomitant medication).
- *Alternative first choice*: AED recommended when certain patient-related factors (e.g., comorbidity, concomitant medication) or AED-related factors (e.g., interaction potential, contraindications, adverse effect profile) preclude the use of the first-choice AED.

Wherever possible, the newly developed ILAE 2017 classification of seizure types was used [4].

## Recommendations for treatment

### Initial monotherapy in adults and children

#### Focal-onset seizures

Registered and reimbursed treatment options for monotherapy of focal-onset seizures (including focal-to-bilateral seizures) in adults and children in Belgium are carbamazepine, lamotrigine (age ≥ 12 years), levetiracetam (≥ 16 years), oxcarbazepine (≥ 6 years), phenobarbital, phenytoin, primidone, topiramate (≥ 6 years) and valproate (Table 1). Since the 2012 publication, pheneturide was withdrawn [12] and lacosamide was approved for monotherapy of focal-onset seizures (≥ 4 years [8, 9]) but is not reimbursed for this indication. Similarly, gabapentin is approved but not reimbursed for monotherapy of focal-onset seizures (≥ 12 years) (Table 1).

**Table 1:** Reimbursed and non-reimbursed indications and main characteristics of antiepileptic drugs (ATC code N03) registered in Belgium

Product name	Reimbursed and non-reimbursed indications^a^	Route	Side effects^b^	Pharmacokinetic interactions^c^	Caution in patients with^d^
Brivaracetam	R: focal-onset, add-on after failure ≥ 3 AEDs, age ≥ 4 y NR: focal-onset, add-on, age ≥ 4 y	p.o. + i.v	≥ 1/10: dizziness, somnolence, behavioral changes (children) Selected: suicidal thoughts	0	Liver impairment, pregnancy (data lacking)
Carbamazepine	R: focal-onset, generalized-onset [GTCS], mono + add-on, any age	p.o	≥ 1/10: dizziness, ataxia, somnolence, fatigue, nausea, vomiting, allergic dermatitis, urticaria, leukopenia, elevated γ-GT (usually not clinically relevant) Selected: serious skin reactions/hypersensitivity (SJS, TEN, DRESS), blood dyscrasia, osteoporosis, osteomalacia, behavioral changes (suicidal thoughts), liver dysfunction, AV block	+ + +	Older age, history of heart, liver or kidney disease, history of drug-induced hematological reactions or oxcarbazepine/phenytoin-induced hypersensitivity, elevated intra-ocular pressure, mixed seizures, pregnancy (teratogen) Contra-indications: AV block, history of bone marrow depression or hepatic porphyria, combination with MAO inhibitors
Ethosuximide	R: generalized-onset [absence, atonic, myoclonic], mono + add-on, age ≥ 3 y	p.o	≥ 1/10: none Selected: abdominal discomfort, serious skin reactions/hypersensitivity (SJS, DRESS), SLE, blood dyscrasia, suicidal thoughts, liver and kidney dysfunction, psychosis	0	Liver and kidney disease, mixed seizures, pregnancy (teratogen)
Felbamate	R: Lennox–Gastaut (if refractory to all relevant AEDs), add-on, age ≥ 4y	p.o	≥ 1/10: none Selected: serious skin reactions/hypersensitivity (SJS, anaphylaxis), severe blood dyscrasia (incl.[aplastic] anemia: 30% fatal), severe hepatotoxicity (30% fatal), suicidal thoughts	+ + +	Pregnancy (data lacking) Contra-indications: history of blood dyscrasia, liver disease
Gabapentin	R: focal-onset, add-on, age ≥ 6 y NR: focal-onset, mono, age ≥ 12 y	p.o	≥ 1/10: dizziness, ataxia, somnolence, fatigue, fever, viral infection Selected: weight gain, serious skin reactions/hypersensitivity (DRESS, anaphylaxis), suicidal thoughts, acute pancreatitis, respiratory depression	0	Use of opioids or other CNS depressants, underlying respiratory condition, older age, renal failure, neurological disease, history of drug abuse, mixed seizures, pregnancy (teratogen)
Lacosamide	R: focal-onset, add-on after failure ≥ 3 AEDs, age ≥ 4 y NR: focal-onset, mono + add-on, age ≥ 4y	p.o. + i.v	≥ 1/10: dizziness, headache, nausea, diplopia Selected: prolonged PR interval, AV block, suicidal thoughts	0	Underlying proarrhythmic conditions, treatment with compounds affecting cardiac conduction, older age, pregnancy (data lacking) Contra-indications: 2nd or 3rd degree AV block
Lamotrigine	R: focal-onset, generalized-onset [GTCS], mono, age ≥ 12 y, add-on, age ≥ 2 y R: absence, mono + add-on, age ≥ 2 y NR: Lennox–Gastaut, mono, age ≥ 13 y, add-on, age ≥ 2 y	p.o	≥ 1/10: headache, rash Selected: serious skin reactions/hypersensitivity (SJS, TEN, DRESS), hemophagocytic lymphohistiocytosis, osteoporosis, suicidal thoughts, arrhythmogenic ST-T abnormality, Brugada ECG, insomnia, hallucinations	+	History of rash or allergy to other AEDs, bipolar and other psychiatric disorders, kidney failure, Brugada syndrome, myoclonic seizures
Levetiracetam	R: focal-onset, mono, age ≥ 16 y, add-on, age ≥ 1 m R: myoclonic in JME, add-on, age ≥ 12y NR: generalized-onset [GTCS] in patients with IGE, add-on, age ≥ 12 y	p.o. + i.v	≥ 1/10: somnolence, headache Selected: acute kidney injury, blood dyscrasia, suicidal thoughts, behavioral changes	0	Kidney or severe liver dysfunction
Oxcarbazepine	R: focal-onset, mono + add-on, age ≥ 6 y	p.o	≥ 1/10: dizziness, somnolence, fatigue, headache, nausea, vomiting, diplopia Selected: severe acute hypersensitivity (SJS, TEN, anaphylaxis), hyponatremia, cardiac conduction defects, liver dysfunction, hypothyroidy, blood dyscrasia, osteoporosis, suicidal thoughts	+	Heart, liver or kidney disease, history of carbamazepine-induced hypersensitivity, treatment with sodium-lowering drugs
Perampanel	R: focal-onset, add-on after failure ≥ 3 AEDs, age ≥ 12 y NR: focal-onset, generalized-onset [GTCS] in patients with IGE, add-on, age ≥ 12 y	p.o	≥ 1/10: dizziness, somnolence Selected: serious skin reactions/hypersensitivity (DRESS), behavioral changes, suicidal thoughts	+	History of drug abuse, severe liver or kidney dysfunction, pregnancy (data lacking)
Phenobarbital	R: focal-onset, generalized-onset (except absence), mono + add-on, any age R: absence, add-on, any age	p.o. + i.v	Dizziness, ataxia, somnolence, nausea, vomiting, headache, visual impairment, nystagmus, diplopia^e^ Selected: serious skin reactions/hypersensitivity (SJS, TEN, DRESS), blood dyscrasia, osteoporosis, osteomalacia, kidney disease, hepatic encephalopathy, addiction, behavioral/cognitive changes, suicidal thoughts, connective tissue disorders	+ + +	Older age, alcoholism, kidney, liver and lung disease, depression, history of drug abuse, pregnancy (teratogen) Contraindications: hypersensitivity to barbiturates, porphyria, (severe) respiratory insufficiency, severe liver or kidney dysfunction
Phenytoin	R: focal-onset, generalized-onset [GTCS], second-line mono + add-on, any age Never for absence	p.o	≥ 1/10: gingival hyperplasia or hypertrophy, somnolence, ataxia, fatigue, diplopia, nystagmus, dysarthria^e^ Selected: serious skin reactions/hypersensitivity (SJS, TEN, DRESS), SLE, hepatotoxicity, lymphadenopathy, osteoporosis, osteomalacia, megaloblastic anemia, blood dyscrasia, absences, myoclonic seizures, suicidal thoughts, cerebellar atrophy, peripheral polyneuropathy, AV block	+ + +	Liver and kidney disease, mixed seizures, pregnancy (teratogen) Contraindications: blood dyscrasia, sinus bradycardia, sinoatrial block, 2^nd^ and 3^rd^ degree AV block, heart failure, Adams–Stokes syndrome, history of hypersensitivity to aromatic anticonvulsants, acute intermittent porphyria
Pregabalin	R: focal-onset, add-on, age ≥ 18 y	p.o	≥ 1/10: dizziness, somnolence, headache Selected: hypersensitivity (angio-edema), blurred vision, kidney failure, congestive heart failure, suicidal thoughts, constipation, addiction, encephalopathy	0	Older age, diabetes, heart disease, kidney dysfunction, history of drug abuse
Primidone	R: focal-onset, generalized-onset [e.g., GTCS, myoclonic, atonic], mono + add-on, any age R: absence, add-on, any age	p.o	Dizziness, ataxia, somnolence, nausea, visual impairment, nystagmus^e^ Selected: serious skin reactions/hypersensitivity (SJS, TEN), addiction, suicidal thoughts, osteoporosis, osteomalacia, megaloblastic anemia	+ + +	Older age, children, liver, kidney or respiratory impairment, history of drug abuse, mixed seizures, pregnancy (teratogen) Contraindications: acute intermittent porphyria, hypersensitivity to phenobarbital
Rufinamide	R: Lennox–Gastaut (if clinical and EEG-based diagnosis and failure ≥ 2 AEDs incl. valproate and topiramate/lamotrigine), add-on, age ≥ 4 y NR: Lennox–Gastaut, add-on, age ≥ 1 year	p.o	≥ 1/10: dizziness, somnolence, fatigue, headache, nausea, vomiting Selected: serious skin reactions/hypersensitivity (SJS, DRESS), induction of seizures/status epilepticus, reduced QT interval, suicidal thoughts	+	Liver dysfunction, increased risk of short QT interval, pregnancy (data lacking)
Stiripentol	R: Dravet, add-on with valproate and clobazam if insufficiently controlled by valproate and clobazam, children	p.o	≥ 1/10: ataxia, somnolence, hypotonia, dystonia, insomnia, anorexia, reduced appetite, weight loss Selected: neutropenia	+ +	Liver or kidney dysfunction, pregnancy (data lacking) Contraindications: history of delirium
Tiagabine	R: focal-onset, add-on, age ≥ 12 y	p.o	≥ 1/10: dizziness, tremor, somnolence, fatigue, depression, nervousness, concentration problems, nausea Selected: induction of seizures/status epilepticus, visual impairment, ecchymosis, behavioral changes, suicidal thoughts	0	History of behavioral disorders, pregnancy (data lacking) Contraindications: severe liver dysfunction
Topiramate	R: focal-onset, generalized-onset [GTCS], mono, age ≥ 6 y, add-on, age ≥ 2 y R: Lennox–Gastaut, add-on, age ≥ 2 y	p.o	≥ 1/10: dizziness, somnolence, fatigue, paresthesia, depression, nausea, diarrhea, weight loss Selected: oligohydrosis, hyperthermia, kidney stones, acute myopia with secondary angle closure glaucoma, visual impairment, metabolic acidosis, hyperammonemia (with/without encephalopathy), cognitive deterioration, suicidal thoughts	+	Liver or kidney dysfunction, increased risk of kidney stones or metabolic acidosis, history of eye disorders, pregnancy (fetal growth, teratogen)
Valproate	R: focal-onset, generalized-onset [GTCS, absence, atonic, myoclonic], mono + add-on, any age	p.o. + i.v	≥ 1/10: tremor, nausea, vomiting, abdominal pain, hyperammonemia Selected: weight gain, hepatotoxicity, pancreatitis, mitochondrial disease (exacerbation), suicidal thoughts, polycystic ovaries, hyperammonemic and non hyperammonemic encephalopathy, thrombocytopenia, osteoporosis	+ +	Hemorrhagic diathesis, AIDS, diabetes, CPTII deficiency Contraindication: acute/chronic hepatitis, (family) history of hepatitis or pancreas impairment, hepatic porphyria, clotting disorders, mitochondrial disease, urea cycle disorders, women of childbearing potential, pregnancy (teratogen, neurodevelopment)
Vigabatrin	R: focal-onset, last-choice add-on, any age R: West, mono, children	p.o	≥ 1/10: somnolence, fatigue, (irreversible) visual field defects, arthralgia Selected: encephalopathic symptoms, movement disorders, psychiatric problems, suicidal thoughts	0	History of visual field defects or behavioral problems/depression/psychosis, older age, kidney impairment, myoclonic and absence seizures, pregnancy (data lacking)

*add-on* add-on therapy, *AED* antiepileptic drug, *AIDS* acquired immune deficiency syndrome, *ATC* Anatomical Therapeutic Chemical (Classification System), *AV* atrioventricular, *CNS* central nervous system, *CPTII* carnitine palmitoyltransferase II, *DRESS* drug reaction with eosinophilia and systemic symptoms, *ECG* electrocardiogram, *EEG* electroencephalogram, focal-onset refers to focal-onset seizures with or without focal-to-bilateral tonic–clonic seizures, *γ-GT* gamma-glutamyl transferase, *GTCS* generalized-onset tonic–clonic seizures, *IGE* idiopathic generalized epilepsy, *i.v.* intravenous, *JME* juvenile myoclonic epilepsy, *m* months of age, *MAO* monoamine oxidase, *mono* monotherapy, *NR* non-reimbursed indication, *p.o.* per os, *R* reimbursed indication, *SJS* Stevens–Johnson syndrome, *SLE* systemic lupus erythematosus, *TEN* toxic epidermal necrolysis, *y* years of age

^a^Information retrieved from each AED’s summary of product characteristics (SmPC) and [25]

^b^Side effects taken from each AED’s SmPC: “ ≥ 1/10” are those listed as occurring at a frequency of 1/10 or higher in clinical trials or observational studies; “selected” are those listed in Sect. 4.4 “Special warnings and precautions for use”, as well as a small number listed in Sect. 4.8 “Side effects”

^c^Information based on SmPCs and author consensus; the risk of pharmacokinetic interactions is graded from 0 (no or minimal risk) to +  +  + (high risk)

^d^Taken from each AED’s SmPC, Sect. 4.4 “Special warnings and precautions for use” and Sect. 4.3 “Contraindications”. Information on teratogenicity complemented with [24, 85]

^e^For phenobarbital, the frequency of adverse effects is not provided in the SmPC; for phenytoin, the listed CNS symptoms have an unknown frequency in the SmPC; for primidone, no recent clinical information is available for an accurate determination of the frequency of adverse effects

Of these options, carbamazepine, lamotrigine, levetiracetam and oxcarbazepine are recommended as first choice, while topiramate and valproate are suitable alternative first choices (except for valproate in women/girls of childbearing potential) (Table 2) [20–23, 26, 27]. Since approximately 50% of patients respond to the first monotherapy [28], it should be appropriately chosen including tolerability profile, risk for future pregnancies and drug interactions. Hence, phenytoin and phenobarbital should not be first choices, despite being approved and reimbursed (author consensus). Evidence from randomized trials is more limited in children [29] and the aforementioned age restrictions should be taken into account in the pediatric recommendations (e.g., levetiracetam is not licensed as monotherapy in children < 16 years). Compared to carbamazepine, which has been used for over 50 years, several of the newer AEDs (e.g., lamotrigine, levetiracetam and oxcarbazepine) have a more favorable pharmacokinetic profile and lamotrigine is better tolerated [30].

**Table 2:** Recommendations for initial monotherapy and add-on therapy for seizures in adults and children

Seizure type	Monotherapy First choice	Monotherapy Alternative first choice	Add-on therapy	Remarks
Focal-onset seizures (including focal-to-bilateral tonic–clonic seizures)	Carbamazepine Lamotrigine (≥ 12 y) Levetiracetam (≥ 16 y) Oxcarbazepine (≥ 6 y)	Topiramate (≥ 6 y) Valproate	Brivaracetam (≥ 4 y) Carbamazepine Gabapentin (≥ 6 y) Lacosamide (≥ 4 y) Lamotrigine (≥ 2 y) Levetiracetam (≥ 1 m) Oxcarbazepine (≥ 6 y) Perampanel (≥ 12 y) Pregabalin (≥ 18 y) Tiagabine (≥ 12 y) Topiramate (≥ 2 y) Valproate	Lamotrigine and levetiracetam are preferred over carbamazepine in older patients due to their better tolerability and lower risk of drug–drug interactions [80, 81] Tiagabine is rarely used; gabapentin and pregabalin are infrequently used Brivaracetam, lacosamide and perampanel are only reimbursed after failure of at least three AEDs Clobazam can be considered as add-on treatment (but is not reimbursed for epilepsy) [19, 22] Vigabatrin can also be used as add-on treatment but only as last choice because of its unfavorable safety profile Avoid valproate in pregnant women/women of childbearing potential
Generalized-onset tonic–clonic seizures, (tonic and atonic seizures)	Valproate	Carbamazepine Lamotrigine (≥ 12 y) Topiramate (≥ 6 y)	Carbamazepine Lamotrigine (≥ 2 y) Levetiracetam (NR; ≥ 12 y) Topiramate (≥ 2 y) Valproate	Carbamazepine can be considered (for tonic–clonic seizures) but should be avoided if absence or myoclonic seizures are present or JME is suspected [22, 23] Lamotrigine can aggravate myoclonic seizures [22] Levetiracetam is also effective as monotherapy for generalized-onset seizures [27, 31, 32] but is currently only licensed as add-on for this seizure type Clobazam could be considered as add-on treatment (not reimbursed) [22] Avoid valproate in pregnant women/women of childbearing potential
Absence seizures	Ethosuximide (≥ 3y) Valproate	Lamotrigine (≥ 2 y)	Ethosuximide (≥ 3 y) Lamotrigine (≥ 2 y) Valproate	Carbamazepine, gabapentin, oxcarbazepine, phenobarbital, phenytoin, pregabalin, tiagabine and vigabatrin may aggravate absence seizures [22, 23] Clobazam and clonazepam could be considered as add-on treatment (not reimbursed) [22] Avoid valproate in pregnant women/women of childbearing potential
Myoclonic seizures	Valproate		Levetiracetam (≥ 12 y; in JME) Valproate	Carbamazepine, gabapentin, lamotrigine, oxcarbazepine, phenytoin, pregabalin, tiagabine and vigabatrin may aggravate myoclonic seizures [22, 23] Clobazam and clonazepam could be considered as add-on treatment (not reimbursed) [22] Avoid valproate in pregnant women/women of childbearing potential

First choice refers to first treatment choice in a patient without any specific factors precluding the use of this antiepileptic drug (AED). Alternative first choice refers to AEDs recommended when certain patient- or AED-related factors preclude the use of the first-choice AED

The efficacy of older AEDs in add-on treatment is considered to be established during long-term clinical experience

*AED* antiepileptic drug, *JME* juvenile myoclonic epilepsy, *m* month of age, *NR* AED not reimbursed for the specified treatment type/indication, *y* years of age

#### Generalized-onset seizures

Generalized-onset seizures include motor seizures (e.g., tonic–clonic, myoclonic, atonic) and non-motor or absence seizures [4]. Not all AEDs indicated for generalized-onset seizures cover all these seizure types. Registered and reimbursed treatment options for monotherapy of generalized-onset tonic–clonic seizures in adults and children in Belgium are carbamazepine, lamotrigine (age ≥ 12 years), phenobarbital, phenytoin, primidone, topiramate (≥ 6 years) and valproate (Table 1). Since the 2012 publication, no new AEDs have become available as monotherapy for this indication in Belgium. Ethosuximide (≥ 3 years), lamotrigine (≥ 2 years) and valproate are registered and reimbursed as monotherapy for absence seizures (Table 1).

Valproate, phenobarbital and primidone are available for the treatment of myoclonic and/or other generalized seizure types (e.g., atonic).

For generalized-onset tonic–clonic seizures, valproate is recommended as first choice (except in women/girls of childbearing potential); lamotrigine and topiramate are valuable alternatives, however, lamotrigine can aggravate myoclonic seizures (e.g., in patients with Juvenile Myoclonic Epilepsy [JME]) (Table 2) [21–23, 27, 31, 32]. Levetiracetam is effective as monotherapy for generalized-onset tonic–clonic seizures [27, 31, 32] but is currently not reimbursed for this seizure type in Belgium. Carbamazepine can also be considered as monotherapy for generalized-onset tonic–clonic seizures but is not effective against other generalized-onset seizure types and can aggravate absences, myoclonic and tonic/atonic seizures [22, 23].

For absence seizures, ethosuximide and valproate are recommended as first choice (except for valproate in women/girls of childbearing potential) and lamotrigine as alternative first choice (Table 2) [20–23, 29, 31, 33]. For myoclonic and tonic/atonic seizures, valproate is recommended as first choice (except in women of childbearing potential) [22, 34]. Levetiracetam could be considered as alternative for myoclonic seizures [22, 34], although it is not registered for monotherapy for this seizure type (Table 2).

Notably, carbamazepine, gabapentin, oxcarbazepine, phenobarbital, phenytoin, pregabalin, tiagabine and vigabatrin may trigger or exacerbate myoclonic and/or absence seizures (Table 2) [22, 23].

#### Seizures of unknown onset

If the seizure type has not been established before starting AED treatment, a broad-spectrum AED (effective against the most common seizure types, such as valproate, lamotrigine, levetiracetam or topiramate) can be used (author consensus).

### Add-on treatment in adults and children

If the AED chosen for initial monotherapy is ineffective in controlling seizures, a second AED is typically selected for either substitution monotherapy (preferred option) or add-on therapy (Fig. 1) [21, 22, 35]. Failure of a second AED may require additional trials of various combination therapies [21, 22, 35]. The choice of add-on AEDs depends on the same compound- and patient-related factors as those impacting the choice of the initial AED, and on the potential for interactions between the compounds. An increasing number of new AEDs, with different mechanisms of action, better pharmacokinetic profiles and better tolerability compared to the older AEDs have been licensed for add-on therapy in the last decades [36, 37]. “Rational polytherapy” relies on combining AEDs with complementary mechanisms of action that may work synergistically to maximize efficacy and minimize toxicity. Clinical trials and observational studies have shown that combinations of drugs with the same mechanism of action (e.g., sodium channel blockers) are typically associated with higher rates of adverse events and treatment discontinuation [35, 38]. Clinical studies have shown synergistic interactions in terms of efficacy for the combination of valproate and lamotrigine, while for other combinations clinical evidence is limited [35, 38]. Therefore, the choice of AEDs in add-on therapy is still made on a case-by-case basis.

#### Focal-onset seizures

Registered and reimbursed AEDs for add-on treatment of focal-onset seizures in adults and children in Belgium are brivaracetam (age ≥ 4 years), carbamazepine, gabapentin (≥ 6 years), lacosamide (≥ 4 years), lamotrigine (≥ 2 years), levetiracetam (≥ 1 month), oxcarbazepine (≥ 6 years), perampanel (≥ 12 years), phenobarbital, phenytoin, pregabalin (≥ 18 years), primidone, tiagabine (≥ 12 years), topiramate (≥ 2 years), valproate and vigabatrin (last choice) (Table 1).

Changes in registration or reimbursement status of drugs used as add-on therapy for focal-onset seizures after the 2012 recommendations include the registration of brivaracetam and perampanel [10, 11], an extension of the lower age limit for lacosamide (from 16 to 4 years [8]) and the withdrawal of pheneturide and retigabine [12, 13].

AEDs recommended for add-on therapy of focal-onset seizures are brivaracetam, carbamazepine, gabapentin, lacosamide, lamotrigine, levetiracetam, oxcarbazepine, perampanel, pregabalin (adults only), tiagabine, topiramate and valproate (except for women/girls of childbearing potential for the latter) (Table 2) [19, 21, 22, 29, 39–42]. The benzodiazepine clobazam can also be considered in patients 6 years or older (but is not reimbursed for epilepsy treatment) [19, 22]. Brivaracetam, lacosamide and perampanel are only reimbursed after failure of at least three AEDs.

#### Generalized-onset seizures

Registered and reimbursed AEDs for add-on treatment of generalized-onset seizures in adults and children in Belgium did not change since the 2012 recommendations; reimbursed compounds are carbamazepine, ethosuximide (≥ 3 years), lamotrigine (≥ 2 years), levetiracetam (for JME, ≥ 12 years), phenobarbital, phenytoin, primidone, topiramate (≥ 2 years) and valproate (Table 1). In addition, levetiracetam and perampanel are registered but not reimbursed for add-on treatment of generalized-onset tonic–clonic seizures in individuals ≥ 12 years.

Lamotrigine, topiramate and valproate are recommended as add-on treatment for generalized-onset tonic–clonic seizures in adults and children (taking into account the aforementioned age restrictions); levetiracetam and clobazam are also options (but are not reimbursed for this indication) (Table 2) [19, 21, 22]. Carbamazepine can be considered for generalized-onset tonic–clonic seizures (but should be avoided with other generalized seizure types). Recommendations for other generalized-onset seizure types are summarized in Table 2.

### Childhood epilepsy syndromes

Table 3 summarizes treatment options in selected epilepsy syndromes in children: simple febrile seizures [43, 44], childhood absence epilepsy [33, 45], childhood epilepsy with centro-temporal spikes [22, 23, 43], electrical status epilepticus during sleep [46], West syndrome [22, 43, 44, 47, 48], Dravet syndrome [49], Lennox–Gastaut syndrome [50] and neonatal-onset epilepsies related to pathogenic variants in potassium and sodium channel genes *KCNQ2, SCN2A, SCN8A* [51, 52]. For most of these syndromes, referral to a tertiary pediatric epilepsy specialist is recommended.

**Table 3:** Childhood epilepsy syndromes

Syndrome	First line	Second line	Avoid
Simple febrile seizures [43, 44]	None	Valproate Levetiracetam	
Childhood absence epilepsy [33, 45]	Ethosuximide	Valproate (Lamotrigine)	Carbamazepine, gabapentin, oxcarbazepine, phenobarbital, phenytoin, tiagabine and vigabatrin [22, 23]
Childhood epilepsy with centro-temporal spikes (Rolandic epilepsy) [22, 23, 43]	Valproate Levetiracetam Sulthiame^a^	Carbamazepine	
Continuous spikes and waves during slow sleep (CSWS)/Electrical status epilepticus during sleep (ESES) [46]	Levetiracetam Steroids Clobazam		Carbamazepine, oxcarbazepine [22]
West syndrome (infantile spasms) [22, 43, 44, 47, 48]	ACTH/prednisolone + vigabatrin	Topiramate Benzodiazepines Valproate	
Dravet syndrome [49]	Valproate + stiripentol + clobazam	Topiramate Cannabidiol^b^ Fenfluramine^c^ Ketogenic diet	Sodium channel blockers (e.g., carbamazepine)
Lennox–Gastaut syndrome [50]	Valproate Rufinamide (add-on) Clobazam	Cannabidiol^b^ Ketogenic diet	Carbamazepine, oxcarbazepine, phenytoin, tiagabine
*KCNQ2*, *SCN2A*, *SCN8A*-related neonatal-onset epilepsies^d^ [51, 52]	Carbamazepine	Phenytoin	

Recommendations are based on author consensus

^a^Sulthiame is registered and reimbursed for some epilepsy indications in Belgium but is not available on the Belgian market and has to be ordered from abroad

^b^Cannabidiol is currently only available as non-reimbursed magistral preparation in Belgium

^c^Fenfluramine is not yet registered in Europe

^d^Choice of treatment depends on the nature of the mutation (gain-of-function versus loss-of-function)

## Specific considerations

### Drug-resistant epilepsy and the importance of early referral

Around 30% of patients are refractory to AED treatment [53–55]. The ILAE’s consensus definition of drug-resistant (or refractory) epilepsy is “failure of adequate trials of two tolerated and appropriately chosen and used AED schedules (whether as monotherapies or in combination) to achieve sustained seizure freedom” [56]. ILAE’s consensus definition may help non-specialists recognize drug-resistant patients and ensure their prompt referral to tertiary care centers for expert evaluation and/or surgery (Fig. 1) [56]. Early referral of patients with drug-resistant epilepsy or with complex epilepsy syndromes (e.g., epileptic spasms [West syndrome], Dravet syndrome, continuous spikes and waves during slow sleep, tuberous sclerosis complex) is paramount to improve patients’ chances to achieve seizure control and avoid impairment of neurological and cognitive development in children, irreversible psychological and social problems, life-long disability and premature death [57, 58]. A recent systematic review showed that a shorter duration of epilepsy was significantly associated with a better seizure outcome after resective epilepsy surgery [59]. Aside from surgery, epilepsy centers can offer specialized diagnostic and therapeutic approaches that may identify the underlying cause of apparent or real drug resistance (e.g., non-adherence to medication, misdiagnosis, inadequate dosing, life style factors) and lead to seizure freedom in patients initially considered drug resistant (Fig. 1) [57, 58].

To treat drug-resistant epilepsy, non-AED treatment options can also be considered, including neurostimulatory approaches (e.g., vagus nerve stimulation [VNS] or deep brain stimulation [DBS]), ketogenic or modified Atkins diet and various complementary and behavioral approaches (Fig. 1) [21, 22, 53, 57, 58]. VNS can be considered in patients who are ineligible for resective surgery or for whom surgery failed. It has been shown to reduce seizure frequency in adults and children, and has shown mild and mostly temporary stimulation-related side effects that are different from common side effects of AEDs [60, 61]. DBS of the anterior nucleus of the thalamus (ANT-DBS) is an alternative neurostimulation modality that has shown long-term efficacy in drug-resistant patients [60, 62]. ANT-DBS has been shown to be well tolerated; most complications are related to the implantation technique. In patients with cognitive decline and mood disorders, caution is warranted [60, 62].

The ketogenic diet is a valid treatment option in children and adults with refractory epilepsy [61, 63]. It should be offered not as a last treatment option but earlier in the treatment flow chart. In some conditions, such as glucose transporter 1 (GLUT1) deficiency, and in some mitochondrial disorders, it is first-line treatment [61, 63]. The ketogenic diet is also increasingly being used in refractory status epilepticus treatment and in some severe inflammatory epilepsies (e.g., new-onset refractory status epilepticus [NORSE]) [63]. In addition, immunotherapy has shown promise as adjunctive treatment in rare cases of autoimmune epilepsy, especially cases of NORSE. In particular, patients with febrile infection-related epilepsy syndrome (FIRES) benefit from targeted immunotherapy with anakinra (an interleukin-1 receptor antagonist) after failure of standard immune therapy such as steroids or intravenous immunoglobulins [64–66].

Cannabidiol has shown efficacy comparable to other AEDs, but only in children with drug-resistant Dravet or Lennox–Gastaut syndrome [67]. Pharmacies in Belgium are now allowed to prepare and dispense medicinal products containing cannabidiol using a magistral formula [68]. In September 2019, one commercial cannabidiol-containing drug (*Epidyolex*, GW Pharma [International] B.V.) was approved by the European Medicines Agency as add-on therapy of seizures associated with Dravet or Lennox–Gastaut syndrome, in conjunction with clobazam, in children ≥ 2 years old [69]. This drug is not yet available in Belgium and magistral preparations are not reimbursed.

### Pharmacokinetic properties and pharmacokinetic interaction profile

Given that many epilepsy patients require life-long treatment, take more than one AED and may take contraceptives or other drugs for diseases related or unrelated to epilepsy, it is important to minimize the risk of pharmacokinetic interactions when selecting AEDs. Pharmacokinetic interactions may alter plasma concentrations of AEDs and other drugs by affecting absorption, transport, distribution (plasma protein binding), metabolism and/or renal elimination [70–72]. This may result in reduced efficacy or tolerability.

Several newer AEDs (particularly brivaracetam, gabapentin, lacosamide and levetiracetam, Table 1) have a lower propensity of interactions because they do not induce or inhibit liver enzymes. The older AEDs, particularly hepatic enzyme inducers (e.g., carbamazepine, phenobarbital, phenytoin and primidone) and inhibitors (e.g., valproate) have a higher propensity for pharmacokinetic interactions (Table 1) [70–72].

### Tolerability

Since efficacy of the newer generation AEDs has not improved substantially compared to the older AEDs, tolerability and safety are often the driving factors in selecting the optimal AED for a patient [73]. Common adverse events reported after AED use include dizziness, somnolence, fatigue, headache and gastrointestinal disturbances [27, 73, 74]. Long-term use of some AEDs (most notably, but not exclusively, enzyme inducers) has been associated with reduced bone mineral density and osteoporosis [75, 76]. Therefore, osteodensitometry and other tests of bone metabolism, as well as dietary and lifestyle advice to minimize the risk of osteoporosis are recommended with long-term AED use [21, 22]. Table 1 includes a summary of the most common adverse effects (occurring in more than 10% of patients as listed in the summaries of product characteristics [SmPCs]) and other important adverse events (as listed in the special warnings and precautions for use in the SmPCs). Detailed information can be found in the SmPCs or recent reviews [73, 74].

### Comorbidity

Several central nervous system-related and other diseases, including depression, anxiety, dementia, migraine, heart disease, peptic ulcers and arthritis are more common in epilepsy patients than in the general population [77]. The presence of concomitant disease is an important determinant in the choice of AEDs since certain AEDs are contra-indicated or require special precautions in some of these conditions. More information on this topic can be found in the SmPCs; a summary is presented in Table 1.

### Elderly

Causes for new-onset seizures in people older than 60 years include cerebrovascular diseases, high blood pressure, diabetes and dementia [78, 79]. Because of the high prevalence of these and other comorbid conditions, polypharmacy and a higher likelihood of dose-related and idiosyncratic adverse effects in the elderly population, selecting the optimal AED for elderly patients is challenging.

Focal-onset seizures with impaired awareness are the most common seizure type in people over 60 years [78]. For this seizure type, the first-choice AEDs for monotherapy in elderly patients are lamotrigine and levetiracetam; gabapentin and lacosamide can be considered as alternatives (but are not reimbursed as monotherapy in Belgium) [20, 21, 23, 80, 81]. Of note, carbamazepine has a poor tolerability profile in elderly people [78, 80, 81].

### Pregnancy

AED use during pregnancy is of concern since AEDs can be transferred to the fetus via the placenta, which can result in fetal growth restriction, major congenital malformations (e.g., neural tube defects and cardiac anomalies) and impaired cognitive development in the child [24, 82–86]. These risks should be balanced against those associated with uncontrolled seizures (particularly generalized tonic–clonic seizures) [24].

Among all AEDs, prenatal exposure to valproate is associated with the highest risk of major congenital malformations, delayed early cognitive skills and neurodevelopmental disorders (e.g., autistic spectrum disorder) [24]. Despite previous efforts to better inform women about these risks and discourage valproate use in girls and women, information was not sufficiently reaching patients [87, 88]. Therefore, the European Commission issued new legally binding measures in 2018 to avoid in utero exposure to valproate [89]. Valproate is now contraindicated in pregnant women unless there is no other effective treatment available. In girls and women of childbearing potential, valproate can only be used if conditions of a new pregnancy prevention program are met [89]. If valproate is the only effective treatment (which is more likely when treating generalized epilepsies), the dose should be kept as low as possible, because malformation rate is dose-dependent. The malformation rate with a daily dose < 650 mg is comparable to that with high doses of carbamazepine or lamotrigine [83].

Lamotrigine and levetiracetam are first-choice treatment options in pregnant women (and women of childbearing potential) because they carry the lowest risk of major congenital malformations and have no known impact on neurobehavioral development (although data for levetiracetam are still limited) [24]. Carbamazepine is recommended as alternative first choice; it shows higher malformation rates than lamotrigine and levetiracetam but no impact on neurodevelopment [24]. Oxcarbazepine could also be considered; malformation rates are low but data on neurobehavioral development are sparse [24].

Importantly, blood drug levels should be monitored because pregnancy can have a major impact on pharmacokinetic properties of AEDs (e.g., altered absorption, increased distribution volume and renal excretion, and induction of hepatic metabolism). Lamotrigine, levetiracetam and oxcarbazepine serum concentrations decline most markedly, and dose adjustments may be necessary during pregnancy and postpartum [24].

### Generic substitution

While generic AEDs are considered bioequivalent with the original brand name products, there are conflicting reports about the effect of switching from brand name to generic or among generic AEDs on seizure control, adverse effects, health care utilization and adherence [90–92]. The Belgian Center for Pharmacotherapeutic Information designates all AEDs as “no switch”, indicating that switching between brands and/or generics is not recommended [5]. When a patient is successfully treated with a particular brand of AED, treatment should be continued with that brand. When initiating treatment, the choice between prescribing a generic or brand-name AED should consider the likelihood of a continuous supply of the compound from the same manufacturer. Importantly, supply problems are a reality for several generics in Belgium [93] and may make health care professionals reluctant to prescribe generic AEDs.

### Precision medicine/personalized treatment

In recent years, great progress has been made in identifying genetic causes of epilepsy and understanding the molecular mechanisms underlying its pathophysiology [51, 94]. This has allowed the identification of potential therapeutic targets and has helped select the most effective treatment for individual patients (personalized treatment or precision medicine). Well-established approaches of precision medicine include a ketogenic diet in patients with GLUT1 deficiency, vitamin B6 in pyridoxine-dependent epilepsy, sodium channel blockers (e.g., carbamazepine) in patients with neonatal-onset epilepsy due to mutations in potassium or sodium channel genes (*KCNQ2*, *SCN2A*, *SCN8A*), avoiding sodium channel blockers in Dravet syndrome (characterized by loss-of-function mutations in the sodium channel gene *SCN1A*), and mTOR inhibitors in mTORopathies (e.g., everolimus to treat focal seizures associated with tuberous sclerosis complex) [51, 94]. Importantly, different mutations in the same gene may have opposite effects and require opposite treatment approaches. For instance, sodium channel blockers only work for *KCNQ2* loss-of-function, and *SCN2A* and *SCN8A* gain-of-function mutations [51, 95]. It is, therefore, crucial that treatment strategies are defined after expert multidisciplinary review of pathogenic variants. With an increasing number of targets and drugs being identified, the field of precision medicine is rapidly evolving and may contribute substantially to the advancement of epilepsy treatment.
